# Forager‐mediated cascading effects on food resource species diversity

**DOI:** 10.1002/ece3.9523

**Published:** 2022-11-18

**Authors:** Clara Mendes Ferreira, Melanie Dammhahn, Jana A. Eccard

**Affiliations:** ^1^ Animal Ecology, Institute for Biochemistry and Biology University of Potsdam Potsdam Germany; ^2^ Behavioural Biology, Institute for Neurobiology and Behavioural Biology University of Münster Münster Germany

**Keywords:** coexistence, functional traits, giving‐up density, landscape of fear, perceived predation risk

## Abstract

Perceived predation risk varies in space and time. Foraging in this landscape of fear alters forager‐resource interactions via cascading nonconsumptive effects. Estimating these indirect effects is difficult in natural systems. Here, we applied a novel measure to quantify the diversity at giving‐up density that allows to test how spatial variation in perceived predation risk modifies the diversity of multispecies resources at local and regional spatial levels. Furthermore, we evaluated whether the nonconsumptive effects on resource species diversity can be explained by the preferences of foragers for specific functional traits and by the forager species richness. We exposed rodents of a natural community to artificial food patches, each containing an initial multispecies resource community of eight species (10 items each) mixed in sand. We sampled 35 landscapes, each containing seven patches in a spatial array, to disentangle effects at local (patch) and landscape levels. We used vegetation height as a proxy for perceived predation risk. After a period of three nights, we counted how many and which resource species were left in each patch to measure giving‐up density and resource diversity at the local level (alpha diversity) and the regional level (gamma diversity and beta diversity). Furthermore, we used wildlife cameras to identify foragers and assess their species richness. With increasing vegetation height, i.e., decreasing perceived predation risk, giving‐up density, and local alpha and regional gamma diversity decreased, and patches became less similar within a landscape (beta diversity increased). Foragers consumed more of the bigger and most caloric resources. The higher the forager species richness, the lower the giving‐up density, and alpha and gamma diversity. Overall, spatial variation of perceived predation risk of foragers had measurable cascading effects on local and regional resource species biodiversity, independent of the forager species. Thus, nonconsumptive predation effects modify forager‐resource interactions and might act as an equalizing mechanism for species coexistence.

## Graphical abstract

A novel measure of diversity at giving‐up density (DivGUD) was applied successfully to evaluate cascading effect of landscapes of fear. Under varying risks, foragers shaped the biodiversity of resources at different spatial scales (local and regional). Differences in functional trait preferences by foragers, due to nonconsumptive effects, might act as a coexistence mechanism for the resource species community.

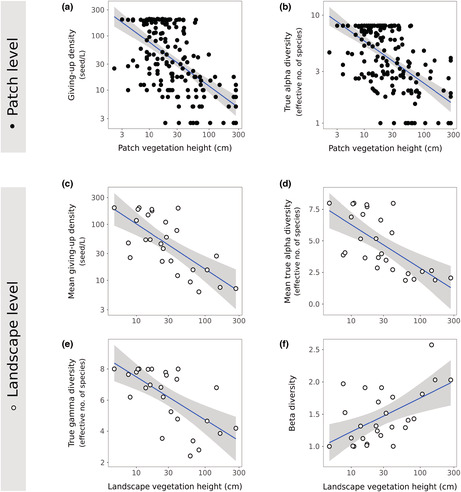

## 1 INTRODUCTION

Complex trophic interactions shape the evolution of plants and animals (Estes et al., 2013; Karban, 2011). In this evolutionary arms race, prey species evolved a set of antipredation strategies such as morphological features (Eklöv & Jonsson, 2007), physiological responses (Boudreau et al., 2019), and behavioral changes such as, for example, reduction in plasticity (Pessarrodona et al., 2019) or the avoidance of predation risk in space and time (Lima & Bednekoff, 1999; Lima & Dill, 1990). Nonconsumptive predation effects cause complex changes in trophic chains, often in the form of top‐down effects (Mitchell & Harborne, 2020). The behavioral responses of prey can be mapped into a landscape of fear, which is defined as the spatiotemporal variation in perceived predation risk by the forager (Gaynor et al., 2019; Laundré et al., 2001, 2014), which affect the distribution of multispecies resources in a landscape (Monk & Schmitz, 2021). The presence of a predator can be evident and perceived directly via sight or smell (Pustilnik et al., 2020; Saavedra & Amo, 2020), or just inferred indirectly by the forager through environmental conditions, such as habitat cover (Wagnon et al., 2020) or variable visibility conditions (Ranåker et al., 2012). Thus, even if no predator is present, foragers perceive predation risk.

Many studies on landscapes of fear focus on predator‐forager interactions and study how the presence/absence of predators can change the morphology, physiology, ecology, and behavior of their prey (Smith et al., 2019). This system can be widened to include forager‐resources interactions into a tri‐trophic system, that is, the interactions among predator‐forager‐resources (Price et al., 1980). These systems allow to study behaviorally mediated trophic cascades of perceived predation risk by the foragers (Smith & Schmitz, 2016), with the nonconsumptive effects of predators affecting the forager's level, consequently changing the population dynamics and multi‐species interactions at the lower trophic level of the resources (Matassa & Trussell, 2011; Mills et al., 2018; Wirsing et al., 2020). The main aim of our study was to zoom in on the consequences of forager‐resource interactions and illuminate how variation in perceived predation risk of foragers elicits cascading effects on the biodiversity of resource species communities at different spatial scales.

Perceived predation risk is often measured using giving‐up density (GUD), which quantifies the resource density left in a patch when the forager decided to quit harvesting (Brown, 1988; Brown & Mitchell, 1989). Since GUD is a measure that depends directly on the forager's feeding behavior under varying perceived predation risk, it became an established method to quantify landscapes of fear (Gaynor et al., 2019; Jacob & Brown, 2000; Van der Merwe & Brown, 2008). Experiments using GUD typically make use of food mixed with a substrate to force the forager to actively search for food in a patch with diminishing returns (e.g., rodents digging in sand trays to find seeds; Brown, 1988; Orrock et al., 2004). If a forager perceives the predation risk as higher, it will quit harvesting sooner as the costs of searching for food surpass the metabolic gains of moving and foraging, the missed opportunity costs, and the predation costs, which results in a higher density of resources left behind when leaving the patch (the GUD). Under the assumption of metabolic and missed opportunity costs being stable, GUD reflects the costs of perceived predation for the forager. Experiments usually make use of a single or few food species (e.g., Brown & Mitchell, 1989) and have a limited ability to assess the top‐down effects of the landscape of fear on the biodiversity of a resource species community.

**FIGURE 1:**
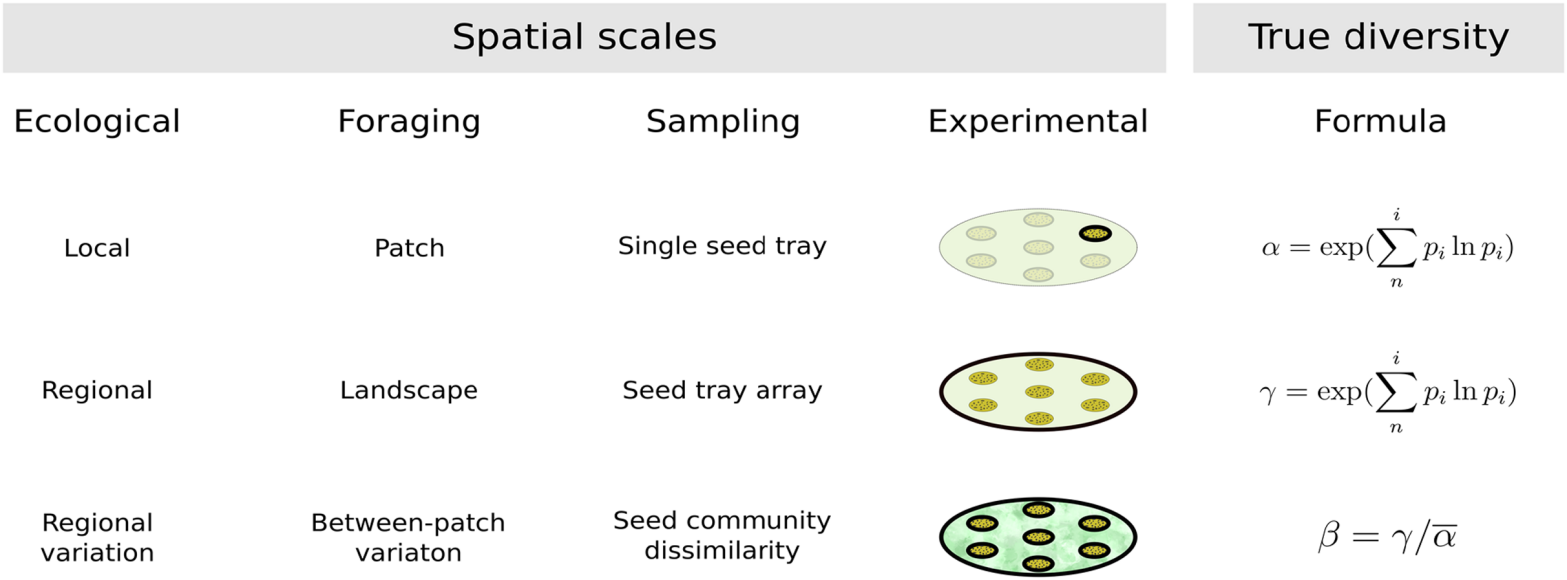
Layout of the experimental design, with the different spatial scales and respective study levels, as well as the true diversities used for each sampling level based on Shannon's entropy and Whittaker's beta diversity (Jost, 2006). Each spatial scale is highlighted with a bold outline in the sketch of the experimental outline.

Using multiple resource species in forager harvesting experiments can, however, further inform of whether variation in foraging can act as a coexistence mechanisms at the resource species level (Garb et al., 2000). Combined with diversity indexes, this approach can illuminate whether and how predation risk effects in prey foraging are a biotic filtering mechanism for biodiversity at the resource level. In this study, we applied a novel measure, the diversity at the giving‐up density (DivGUD; Eccard et al., 2022) and provided a resource species community to foragers and, similarly to GUD, quantified the diversity of resource species left behind in a patch by the foragers. The DivGUD approach can also be used to provide information at different spatial sampling scales since it is measured using classical diversity indices (Whittaker, 1972) on different spatial scales (Figure 1). Species diversity at the local level (foraging patch) is alpha diversity (α‐DivGUD) and is driven by forager‐specific behavior and their individual interactions with the patch at the microhabitat level. When the scale is expanded to contain the cumulative information of several foraging patches, gamma diversity (γ‐DivGUD) at a regional level (foraging landscape) can be assessed. At a landscape, differences in resource species composition among local patches can be assessed as the beta diversity (β‐DivGUD).

Changes in DivGUD might occur due to the dietary preferences of the forager, as predicted by optimal foraging theory (Stephens & Krebs, 1986), as resource species present functional traits (i.e., characteristics that may increase the individual's fitness or performance; McGill et al., 2006), which can also be beneficial for the forager's energy intake. Therefore, foragers are expected to select resources based on expected energetic gains, and change the final relative abundance and species richness of the resource community in a functional‐trait‐dependent way (Eccard et al., 2022). The relevant functional traits of resource species may include morphological traits (e.g., seed size and presence of a husk; Lichti et al., 2017), and physiological traits that increase the competitive capability of resources (e.g., plant nutrients and energy storage correlated to development and growth; Salgado‐Luarte & Gianoli, 2012). Differential feeding by foragers also acts as a biotic filter for resources, creating further variation in population dynamics of the resource species, and contributing further to resource species coexistence as an equalizing mechanism (Chesson, 2000; Larios et al., 2017). We expect perceived predation risk to modify the strength of coexistence mechanisms. Under elevated risk, the foragers ought to consume resources that provide them with the most energetic intake, thus reducing the abundance of resource species that might have a competitive advantage over others (Kotler & Holt, 1989; Stump & Chesson, 2017). Alternatively, when the perceived risk is high, the foragers might be less selective when feeding as they spend less time in the food patch (Eccard et al., 2022).

We used ground‐dwelling rodents as a study system, whose perception of predation risk is often related to how exposed they are in their surrounding habitat. While they react to the olfactory cues of terrestrial carnivores (Eccard et al., 2008; Moll et al., 2020), vegetation cover is their main proxy for the omnipresent and less predictable avian predation risk (Kotler, 1992; Yadok et al., 2019), which translates into a landscape of fear mapped in experiments (e.g., Dammhahn et al., 2022; Eccard et al., 2022; Eccard & Liesenjohann, 2014). Since small rodents are both primary consumers of resources and prey to several secondary consumers, they serve as a suitable connector in a tri‐trophic system model. Small rodents are also important predators of seeds, often shaping plant coexistence in ecosystems (Dylewski et al., 2020; Garb et al., 2000), especially due to their preferences for larger and most caloric seeds (Chang & Zhang, 2014; Mortelliti et al., 2019; Wang & Chen, 2009).

Here we investigated the cascading effects of the landscape of fear on food resource species diversity, using different vegetation heights as a proxy for the perceived predation risk of small rodent foragers (Dammhahn et al., 2022). We provided a resource species community of seeds with different functional traits (size, caloric content, and husk) in discrete food patches to wild foragers. We assessed GUD and DivGUD on the resource level at two different foraging scales (patches—α‐diversity, and landscapes—γ‐diversity and β‐diversity; Eccard et al., 2022) to test the following predictions:

- With increasing vegetation height, i.e., decreasing perceived predation risk, both α‐diversity and γ‐diversity of food resource species would decrease, as the foragers stay longer in the patch and target single, highly rewarding food species.
- We expected β‐diversity of food resource species to increase with vegetation height, as microhabitat heterogeneity should increase with vegetation height, which might impact the presence and foraging behavior of rodents.
- The removal of each resource species should not be at random under varying risk but related to how much it is preferred by the forager due to size and nutritional value, with bigger and/or most nutritious seeds being removed first, independent of vegetation height (perceived risk).
- To account for the confounding effects of working with a whole community of wild rodents, we also assessed how forager species richness in the landscape can affect GUD and DivGUD. Assuming that different co‐occurring rodent species experience similar predation risk and have similar food preferences, we expected them to react similarly to perceived predation risk and, thus, effects of vegetation height on diversity measures being independent of the identity or diversity of forager species.

## 2 MATERIALS AND METHODS

### 2.1 Study site

We conducted a landscape‐wide experiment at the Ecological Research Station Gülpe, in Brandenburg, Germany (52°44′00.1″N 12°12′41.7″E). The study area is characterized by a mixture of grassland and extensively used grasslands (Burkart et al., 2003). Meadows are mowed twice a year, so small to medium (2–50 cm) grass species are dominant, representing around 80% of the area. Riparian corridors with shrubs and reeds (50–280 cm) cover around 20% of the area. The area harbors a diverse community of small rodents (Kath, 2012), with the possible occurrence of four murine species (*Apodemus agrarius*, *A. flavicollis*, *Micromys minutus*, *Mus musculus*) and four vole species (*Arvicola terrestris*, *Microtus agrestris*, *M. arvalis*, *Myodes glareolus*).

### 2.2 Experimental set up

The sampling was done in autumn, for three consecutive nights, in a total of 35 locations (September 2017: eight locations; December 2018: 17 locations; December 2019: 10 locations) with different vegetation heights (our proxy for perceived predation risk). We chose locations based on their accessibility to pathways, their independence from each other (inter‐landscape distances: median = 227 m, min‐max range: 25–630 m), and also aimed to fill a gradient between 5 and 200 cm of vegetation height. We performed the experiment in autumn to avoid an over‐abundance of natural resources, which would change the metabolic gains and costs, as well as to reduce missed opportunity costs as rodents do not breed after September in the sampled area (Niethammer & Krapp, 1978). Both these costs would create a confounding effect for GUD/DivGUD analyses. Within sampling years, we set up all locations simultaneously to avoid confounding effects of weather and lunar cycles (Kotler et al., 2010; Wróbel & Bogdziewicz, 2015).

At each of the 35 locations, we placed an array of seven foraging patches that were hexagonally distributed with one patch in the center and separated by 6 m between patches (Figure 1). The spatial coverage of the patches at each location was chosen to reflect home range sizes of the naturally occurring rodent species, which are reduced in size during late autumn/winter (Baláž & Ambros, 2012; Briner et al., 2005; Yletyinen & Norrdahl, 2008), and to ensure a variety of microhabitats in each location. Each array of patches covered an area of 113 m^2^, and therein will be referred to as a landscape. We measured the vegetation height in each patch at four random points up to 1 m from the patch and averaged within each patch for patch‐level analyses and across the landscape for landscape‐wide analyses. Vegetation height (varying from 2 to 271 cm) was used as a continuous variable, or, for similarity analysis, converted into three categories, by pooling all the average vegetation heights and using the first and third quantiles as thresholds (Low: ≤15 cm, *n* = 13; Medium: >15 cm and ≤52 cm, *n* = 15; High: >52 cm, *n* = 7). Due to the managed grassland nature of the sampled area, vegetation density (sampled as proportion in 1 m^2^) was highly correlated to vegetation height (Kendall's correlation: *r*_T_ = .65, *p* < .001), therefore, vegetation height could serve as a good proxy for both vegetation cover and density.

Each patch consisted of a plastic tray with 400 ml of fine sand (⌀ = 14.5 cm, depth = 4 cm), mixed with seeds of eight different plant (resource) species: sunflower, kardi, wheat, hemp, flaxseed, millet, canary seed, and sesame (Table 1). Each patch contained 10 seed items of each species, i.e., an initial total of 80 seed items per patch was provided. A protective cover was sheltering the sand from rain, small enough as to provide sufficient shelter from mild rain but not from predators for the foragers. The patches and covers were set up before early afternoon and were monitored for foraging activity at every dawn over three consecutive days, by checking the patches for signs of digging in the sand, droppings, and empty seed husks.

**TABLE 1:** Additional information on the plant species used as resources in the experiment

Seed	Species	Mass per item (mg)	Kcal/100 g	Cal/item	Reference
Sunflower	*Helianthus annuus*	38.8	679	263	U.S. Department of Agriculture (2021)
Kardi	*Carthamus tinctorius*	35.2	517	182	U.S. Department of Agriculture (2021)
Wheat	*Triticum aestivum*	39.4	326	128	Package
Hemp	*Cannabis sativa*	11.9	461	55	Package
Flaxseed	*Linum usitatissimum*	7.0	538	38	Package
Millet	*Pennisetum glaucum*	6.1	384	23	Package
Canary seed	*Phalaris canariensis*	4.6	399	18	CSDCS (2016)
Sesame	*Sesamum indicum*	3.7	600	22	Package

*Note*: Mass per seed item of each species was obtained by weighing 100 seeds and dividing it by 100. Energetic content is based on the package information, or when this information was missing, on external sources (reference). Kcal/item was calculated based on these information.

We obtained information on the diversity of foragers for the sampling period of 2018 and 2019, by setting‐up wildlife cameras pointed directly at the tray to identify the forager's species and their activity before the third night of the experiment. We placed two cameras per visited landscape, by randomly choosing two of the foraged patches. Landscapes with no visits were not surveyed with cameras due to logistic constrains.

At the end of the third night, the trays were collected and dried in an incubator at 60°C to filter the sand easily and recover all remaining intact seeds. The final number of seeds of each provided resource species was counted for each patch. We did not include in the final datasets five patches that were evidently affected by human error (e.g., had complete misses of single resource species, but counts of other species were within normal range), as well two patches not found at the end of the experiment. Furthermore, we also removed data from seven more patches (63–270 cm) that were completely depleted by the foragers, as the GUD/DivGUD measures cannot be calculated from them.

### 2.3 Data analyses

We analyzed our data at the patch level (*n* = 231 patches) and at the landscape level (*n* = 35 landscapes; see Figure 1). At the patch level, we first tested whether the probability of a patch being used (yes/no) was explained by the patch vegetation height, using a generalized linear mixed‐effect model (GLMM) with a binomial error distribution. In this and subsequent models, we normalized the vegetation height variable with a natural logarithm transformation. The landscape identity was used as a random factor (random intercept), to account for potential nonindependence of patches within a landscape, due, for example, to the same foraging individual. Furthermore, we also included year as a fixed effect to control for potential differences among years in this and subsequent models.

We calculated GUD by summing the counts of seeds left in the patch and dividing it by the initial 0.4 L of sand (seed/L) and α‐DivGUD using the formula given in Figure 1. All diversity indices were expressed as true diversities (i.e., effective number of species; Hill, 1973) and calculated based on the exponential of Shannon's entropy (Jost, 2006) using the vegan package in R (Oksanen et al., 2020). We chose to use Shannon's entropy due to its sensitivity to diversity changes and because it is known to be accurate in cases of complete sampling, even though it may weight rarer species disproportionally high (Nagendra, 2002). We used a linear mixed‐effect model (LMM) to test the effect of patch vegetation height on GUD or α‐DivGUD, respectively, with landscape identification included as a random factor. GUD was log‐transformed.

At the landscape level, we summed the species‐specific seed counts from all the patches of each landscape and averaged the vegetation heights of each patch over the landscape (landscape vegetation height). We calculated average GUD across the landscape and γ‐DivGUD based on the cumulative seed counts. To obtain a landscape mean ɑ‐diversity ($\bar{\alpha }$‐DivGUD) we averaged across all α‐DivGUDs of the seven patches. To evaluate the dissimilarity of resource species communities within landscapes, we calculated β‐DivGUD for each landscape by dividing the γ‐DivGUD by $\bar{\alpha }$‐DivGUD (Whittaker, 1972).

We analyzed the differences in resource community composition using analysis of similarity and visualized it with nonmetric multidimensional scaling (NMDS) plots. The dissimilarity of resource diversity in the different vegetation categories was calculated with the adonis function in the vegan package in R with 999 permutations. The NMDS plots were also generated using the vegan package, using the dissimilarity matrices calculated previously with the metaDMS function.

The photos from the wildlife cameras were analyzed using the software Digikam, and for each photo, we labeled the forager's species and the landscape name. We exported all relevant metadata using EXIFTOOL and managed the photo database in EXCEL. The final database contained only photos with the presence of foragers, from which the species could be clearly identified. Eight landscapes with no activity recorded were excluded, as well as nine landscapes where the seed tray was not visible for the entire time due to external conditions or logistical problems (e.g., strong flash, rain droplets, etc.).

We first tested whether forager species richness (as number of rodent species observed per landscape) varied with the landscape vegetation height and/or sampling year, using a generalized linear model (GLM) with a Poisson error distribution. Second, we built a linear regression model to test whether variation in GUD was predicted by the landscape vegetation height and/or forager's species richness, we used similarly structured models to predict variation for each DivGUD spatial level ($\bar{\alpha }$‐DivGUD, γ‐DivGUD, and β‐DivGUD). We checked whether adding the forager's species richness improved the model, using the Akaike's Information Criterion (AIC). We also evaluated the potential effects of spatial autocorrelation on forager species richness with the Moran's I index (Moran, 1950) with the ape package (Paradis & Schliep, 2019).

All analyses were done in R 4.0.4 (R Core Team, 2021). If not specified otherwise, all analyses were run with the lm4 package (Bates et al., 2015). The accepted significance level was set to *α* < .05.

## 3 RESULTS

**FIGURE 2:**
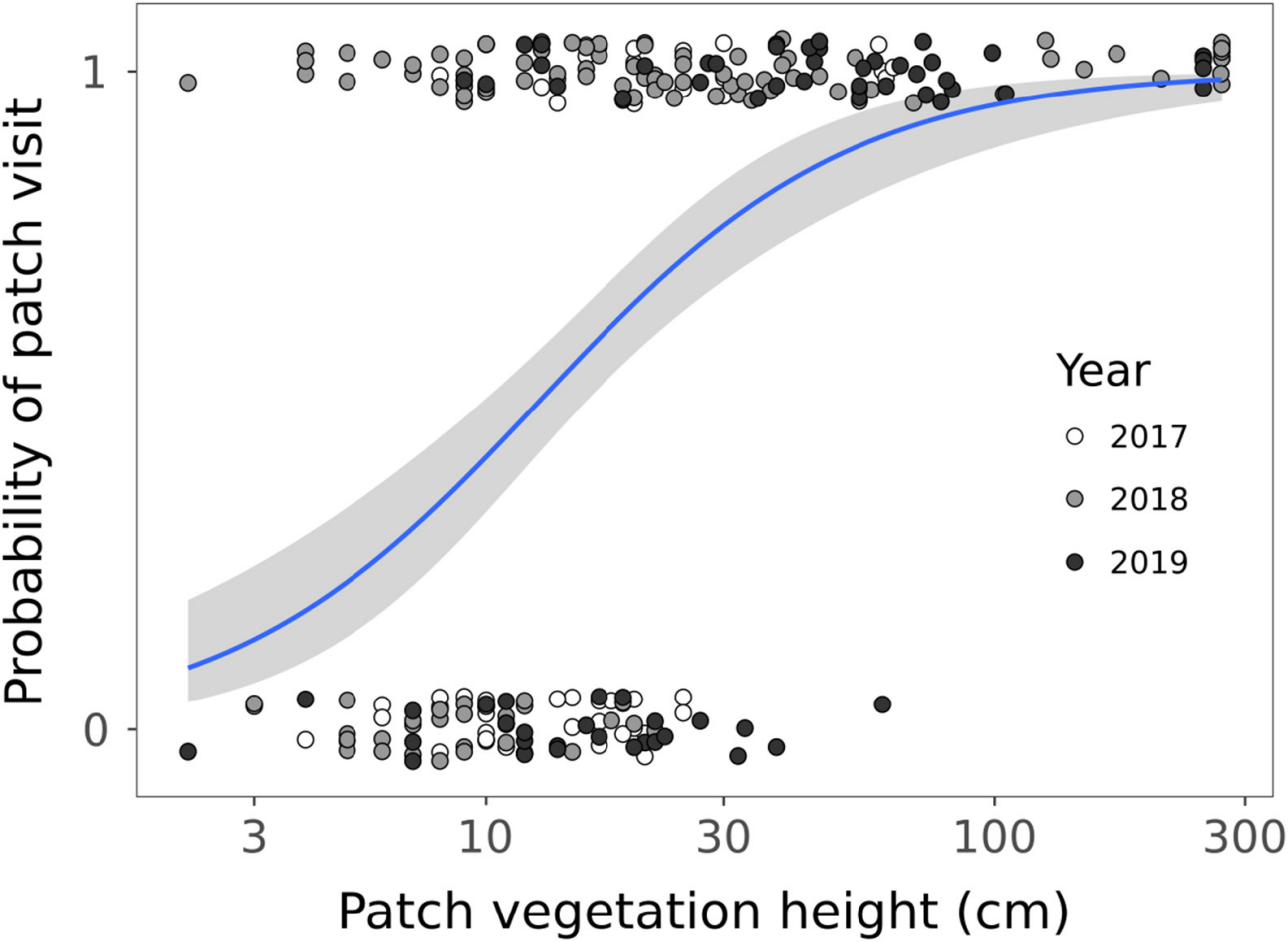
Probability that a forager visited (1) or did not visit (0) a foraging patch in relation to the average vegetation height (logged) at the patch level among the sampled years. The blue trend line and its 95% confidence intervals (gray) are based on a logistic regression without the landscape as a random effect.

At the patch level, the probability of a patch being used increased with vegetation height and between 2017 and 2018 (Table 2, Figure 2). In eight landscapes foragers never visited a single patch (vegetation height: 2–27 cm). These landscapes had scarce vegetation cover and no recent signs of forager presence (e.g., fecal pellets) could be found. With absent foragers, we cannot measure GUD/DivGUD, therefore we removed these landscapes from subsequent analyses. The new datasets included 27 landscapes with a total of 177 patches, in which at least one patch was foraged (i.e., forager presence was confirmed).

**TABLE 2:** Patch level—Results of LMMs on the relationship between the average vegetation height (perceived predation risk) at the patch level, and probability that a forager visited (1) or did not visit (0) a foraging patch (*n* = 231 patches), as well giving‐up density (GUD) and local diversity at the giving‐up density (α‐DivGUD) for *n* = 177 patches (“Vegetation model”), together with differences among the sampled years of 2017–2019. The models included landscape identity as a random effect. Landscape level—Results of linear regression models at the landscape level, above for the “Vegetation model” simple linear regression between the (logged) average vegetation height (of all patches within a landscape) in relation to GUD and DivGUDs, with the complete data from 2017–2019 (*n* = 27 landscapes); and the “Vegetation + Forager model”—multiple linear regression between the average vegetation height and forager's species richness in relation to GUD and DivGUDs, with restricted data from 2018–2019 (*n* = 13 landscapes).

	Patch level						Landscape level							
	Probability of patch visit (0/1)		Logged giving‐up density (GUD, item/L)		True alpha diversity (α‐DivGUD)		Logged mean giving‐up density (mean GUD)		Mean true alpha diversity ($\bar{\alpha }$‐DivGUD)		True gamma diversity (γ‐DivGUD)		Beta diversity (β‐DivGUD)	
	*β* ± SE	*p*	*β* ± SE	*p*	*β* ± SE	*p*	*β* ± SE	*p*	*β* ± SE	*p*	*β* ± SE	*p*	*β* ± SE	*p*
Vegetation model														
Intercept	−7.69 ± 2.23		5.99 ± 0.44		8.86 ± 0.82		6.64 ± 0.58		10.02 ± 1.22		10.47 ± 1.02		0.66 ± 0.25	
Logged average vegetation height (cm)	**2.30 ± 0.65**	**<.001**	**−0.74 ± 0.12**	**<.001**	**−1.24 ± 0.23**	**<.001**	**−0.84 ± 0.17**	**<.001**	**−1.56 ± 0.35**	**<.001**	**−1.24 ± 0.29**	**<.001**	**0.24 ± 0.07**	**.003**
Year (2017–2019)														
2018	**3.74 ± 1.56**	**.016**												
2019	0.85 ± 1.65	.605												
Chisq (df)	7.07 (2)		31.93 (1)		26.30 (1)									
*R* ^2^ marginal	.42		.28		.24									
*R* ^2^ conditional	.82		.67		.67									
Random effect variance	0.76		0.75		2.73									
Fstat (df)							25.90 (1,25)		20.38 (1,25)		18.64 (1,25)		11.28 (1,25)	
multi *R* ^2^							.51		.45		.43		.31	
adj *R* ^2^							.49		.43		.40		.28	
Vegetation + Forager model														
Intercept							7.08 ± 0.66		11.57 ± 1.15		10.92 ± 1.40		0.35 ± 0.38	
Logged average vegetation height (cm)							**−0.75 ± 0.20**	**.004**	**−1.71 ± 0.34**	**<.001**	−0.92 ± 0.42	.053	**0.35 ± 0.11**	**.011**
Forager's species richness							**−0.39 ± 0.15**	**.024**	**−0.59 ± 0.25**	**.044**	**−0.76 ± 0.31**	**.035**	−0.03 ± 0.08	.716
Fstat (df)							15.27 (2,10)		20.39 (2,10)		7.71 (2,10)		5.09 (2,10)	
multi *R* ^2^							.75		.80		.61		.50	
adj *R* ^2^							.70		.76		.53		.41	
Model selection														
Inclusion of year: ∆AIC (*p*)	**−3.07 (.029)**		1.93 (.355)		3.23 (.680)		2.51 (.062)		−1.97 (.422)		2.57 (.061)		−3.37 (.765)	
Inclusion of forager's species richness: ∆AIC (*p*)							**−5.02 (.023)**		**−3.53 (.054)**		**−4.07 (.035)**		1.82 (.716)	
Inclusion of forager's species richness plus year: ∆AIC (*p*)							0.81 (.175)		1.72 (.666)		1.16 (.458)		1.98 (.899)	

*Note*: Shown are the estimated effects (*β*), their standard errors (SE), the *p*‐value as obtained through the R package lmerTest (Kuznetsova et al., 2017), and the *R* ^2^ based on the fixed factors (*R* ^2^ marginal) and based on fixed and random factors (*R* ^2^ conditional). Chi‐square (Chisq) or F‐statistic (Fstat) with degrees of freedom (df), as well the multiple *R* ^2^ (multi *R* ^2^) and adjusted *R* ^2^ (adj *R* ^2^), and change in Akaike's Information Criterion (∆AIC). The sample size of the “Vegetation model” was adjusted to be comparable with the “Vegetation + Forager model” during model selection with the forager's species richness variable. All significant relations are shown in bold.

**FIGURE 3:**
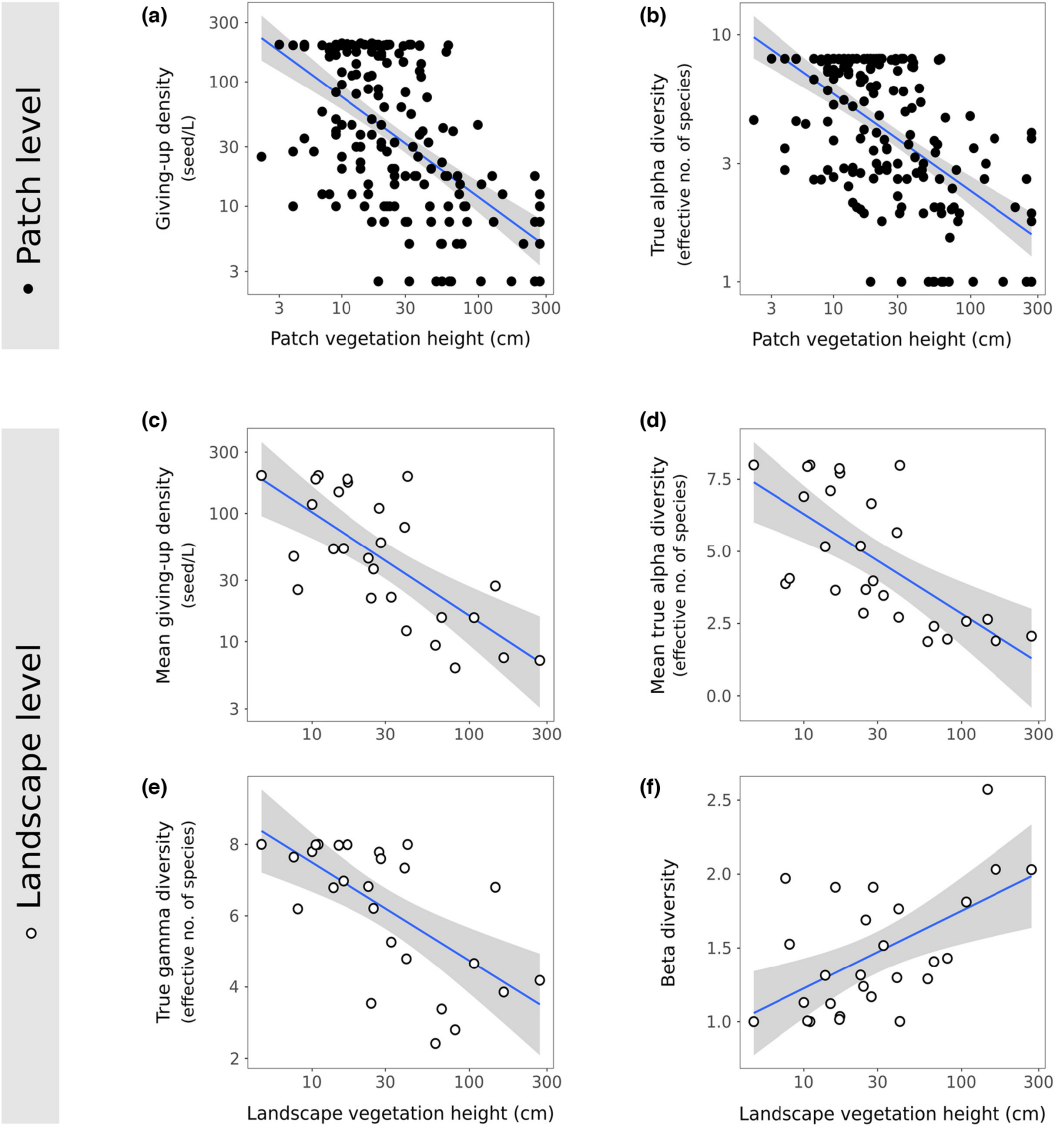
Patch level—relation between average vegetation height at a foraging patch and: (a) giving‐up density (logged) and (b) local diversity at the giving‐up density (α‐DivGUD). Landscape level—relation between the average vegetation height (logged) and: (c) mean giving‐up density (logged) (d) the mean local diversity at the giving‐up density ($\bar{\alpha }$‐DivGUD), (e) the regional diversity at the giving‐up density (γ‐DivGUD), and (f) regional variation ratio (β‐DivGUD). The blue trend lines and their 95% confidence intervals (gray) are based on linear models, without the landscape as a random effect in the “patch level.”

In subsequent models, the inclusion of year as a variable did not improve the models nor was it significant, so this variable was dropped. At the patch level, both GUD (Figure 3a) and α‐DivGUD (Figure 3b) decreased with average vegetation height (Table 2). Similarly, at the landscape level, all GUD (Figure 3c) and DivGUD (Figure 3d,e) measures decreased with average landscape vegetation height (Table 2), except for β‐DivGUD (Figure 3f) that scaled positively with landscape vegetation height.

**FIGURE 4:**
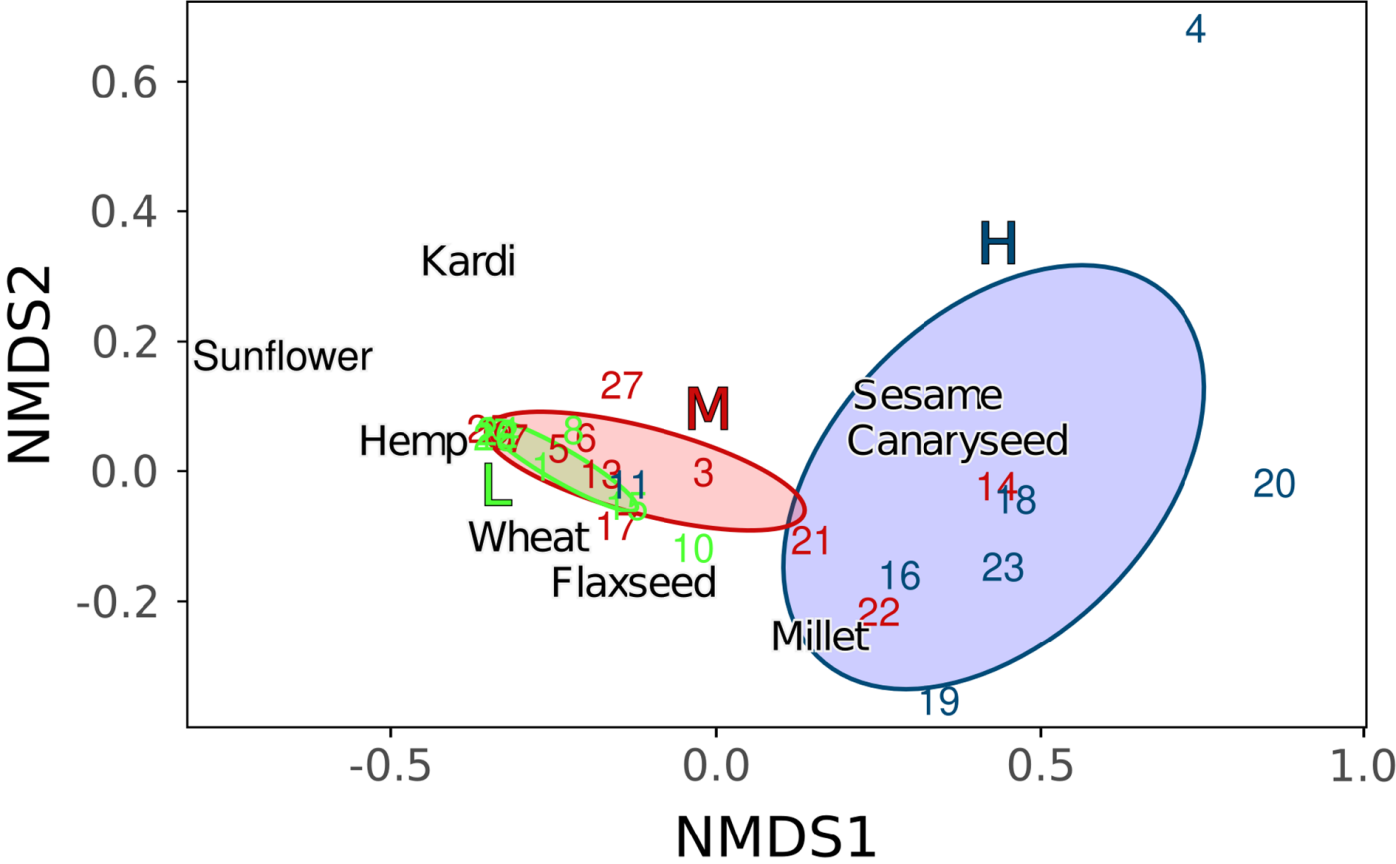
Nonmetric multidimensional scaling (NMDS) of the final resource species' seeds community (true gamma diversity) left over in 27 landscapes (numbers) divided into three categories of vegetation height: Low: up to 15 cm (“L”, green); Medium: from 15 to 52 cm (“M”, red); High: more than 52 cm (“H”, blue). The axes (NMDS1 and NMDS2) can be related to the functional traits of the eight seed species (see Table 1). We used a three dimensions model when generating the plot, as this model converged and presented a low‐stress value (0.03).

Remaining resource species communities were dissimilar among categories of vegetation height (Analysis of similarity: *R* ^2^ = .39, *p* = .001). Graphical inspection of the NMDS plot (Figure 4) suggests that landscapes in the high vegetation height category (“High”: >52 cm) showed a different resource composition from other vegetation height categories (pairwise comparisons; “Low” – “High”: *R* ^2^ = .52, *p* = .002; “Medium” – “High”: *R* ^2^ = .39, *p* = .001), while the low and medium vegetation height categories overlapped (“Low” – “Medium”: *R* ^2^ = .02, *p* = .636). In the high vegetation category, foragers left over a higher proportion of small and less caloric seeds (Figure S1).

The camera surveillance in 2018 and 2019 yielded a total of 1246 photos, and we identified four rodent taxa that foraged in 19 patches of 13 landscapes (out of 35 patches and 19 landscapes kept under surveillance). The most common species was the yellow‐necked mouse (*Apodemus flavicollis*, nine landscapes), followed by *Microtus* spp. (seven landscapes), bank vole (*Myodes glareolus*, six landscapes) and harvest mouse (*Micromys minutus*, three landscapes). *Microtus* voles are difficult to separate into species based on wildlife camera pictures and both common voles (*Microtus arvalis*) and field voles (*M. agrestis*) were previously recorded to be present in the study area (Kath, 2012). Recorded activity (minimum seconds spent on a landscape) was higher for medium to high vegetation heights (Figure S2). We did not record any other nonrodent taxa foraging in our seed trays.

**FIGURE 5:**
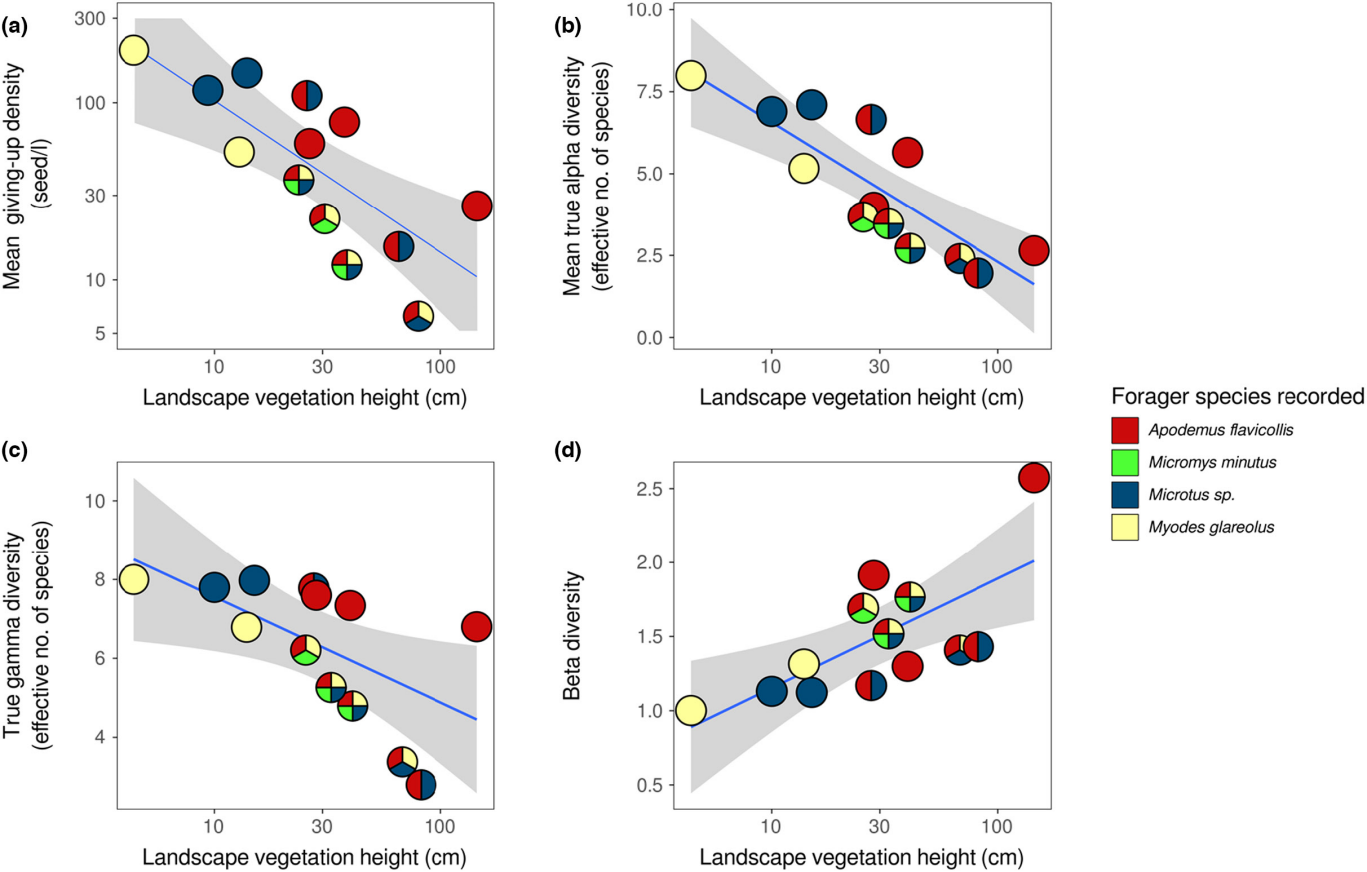
Relation between the average vegetation height (logged) and composition of the forager community, and: (a) the mean giving‐up density (logged); (b) the mean local diversity at the giving‐up density ($\bar{\alpha }$‐DivGUD); (c) the regional diversity at the giving‐up density (γ‐DivGUD); (d) regional variation ratio (β‐DivGUD). The blue trend lines and their 95% confidence intervals (gray) are based on linear models, without the landscape random effect in the “patch level.”

Forager species richness (number of species per landscape) did not vary with average vegetation height (*β* = 0.21 ± 0.24, *p* = .372, residual deviance = 7.41 on 11 df), nor between years (*β* = −0.48 ± 0.45, *p* = .287, residual deviance = 7.01 on 11 df). GUD and $\bar{\alpha }$‐DivGUD decreased with an increase in forager's species richness and average vegetation height in the data from 2018 to 2019 (Table 2, Figure 5). γ‐DivGUD decreased significantly with an increase in forager's species richness, with a decreasing trend when average vegetation height increased. β‐DivGUD increased significantly with increased average vegetation height, with forager's species richness having no effect. All models were improved by the inclusion of forager species richness, except for β‐DivGUD. Forager species richness was not spatially autocorrelated (*I*_2018_ = 0.12, *p* = .106; *I*_2019_ = −0.17, *p* = .512).

## 4 DISCUSSION

Top‐down effects of predators cascade down to the primary resource level and, thus, shape complex processes in ecosystems. Here, we showed under natural conditions that foragers adjust their foraging behavior to the protective cover of vegetation height, with consequences on the biodiversity of the resource species community (prediction i).

At the foraging patch level, the higher the vegetation height, the more resources were exploited by foragers, resulting in lower densities of food when quitting the patch (GUD). This finding was expected based on previous GUD studies, but necessary to confirm that in our experimental landscapes rodents indeed perceived higher predation risk in short vegetation and, thus, variation in vegetation height maps a landscape of fear. The exploitation pattern at the patch level follows the prediction of the patch‐use model (Brown, 1988, 1992) as demonstrated many times, particularly using vegetation height and cover as proxies for perceived predation risk (e.g., Jacob & Brown, 2000; Yadok et al., 2019). Some of the landscapes with very short vegetation were not visited at all. In these cases, it remains to be disentangled whether foragers were completely absent from these areas (which could also be due to high perceived predation risk) or did not visit the food patches with low vegetation because it was too risky to forage in these patches.

In addition to GUD, we could also show that the diversity of resource species left in a patch also decreased with decreasing perceived predation risk. Thus, foragers feeding for longer, more often, or more efficiently in a patch, reduced not only the amount of food left behind but also the local biodiversity (ɑ‐DivGUD). The same pattern occurred at the regional level, with both density and biodiversity (γ‐DivGUD) being lower in landscapes perceived as safer by the foragers. These changes in biodiversity are direct measures of the cascading effects of a forager's landscape of fear and connect variation in the foragers' feeding behavior to changes in ultimate resource species composition.

Contrary to ɑ‐DivGUD and γ‐DivGUD, regional variation between patches (β‐DivGUD) increased with the decrease in perceived predation risk (prediction ii). This pattern was expected, as habitats with higher vegetation heights can also present a greater variety of natural plant diversity, creating the potential for microhabitat effects of variation that may influence foraging (Orrock et al., 2004; Thompson, 1982). In some of these foraging landscapes, we observed that one or two patches were barely used, while the remaining patches were almost depleted. This might have happened because the vegetation cover at the patch level could be variable, even though the maximum vegetation height was still very high. In the patches with high vegetation height, the habitats were not managed as the other grassland areas, which might create further variation in vegetation cover and further influence predation risk (Hinkelman et al., 2012). However, even at more homogenous vegetation height distributions, patches might not have been exploited equally across landscapes (increased β‐DivGUD), as the smaller seeds are difficult to find in the sand and foragers might give up searching at different density and diversity of small seeds. This exploitation pattern can be further observed in the analyses of the final composition of the resource community in higher vegetation heights (prediction iii), as the smaller and less caloric resources (i.e., millet, canary seed, and sesame) were left behind by the foragers at very different densities among foraging patches, and thus increasing the regional variation of resources. Rodents are known to have a preference for larger and/or more nutritious seeds (Eccard et al., 2022; Fischer & Türke, 2016; Wang & Yang, 2014), and it is likely that our foragers extracted those resources first in all foraging patches, rather than randomly selecting seeds, especially at high perceived risk. In the landscapes with high perceived risk, the foragers might limit their time feeding on those seeds, despite potentially higher handling time with larger seeds (Kelrick et al., 1986), and also evenly forage on the patches (low β‐DivGUD). However, in our study, we cannot differentiate the effects of size and energetic content, as most of the larger seeds also contain more calories, and are also encountered first due to their size. Because we can only assess intact seeds left in the tray, we also cannot take into account the feeding strategies of the rodents, namely, if they scatter‐hoard the seeds or not, which can change their preference to lighter seeds that are easier to transport (Muñoz & Bonal, 2008). Independently of which characteristic is favored the most, we could still observe that the rodents forage differently based on the functional traits of the seeds.

Size and energetic content are functional traits that give seeds a competitive advantage at germination and growth (Lichti et al., 2017; Salgado‐Luarte & Gianoli, 2012), but at the same time, these characteristics also make these seeds more profitable food sources for foragers and, thus, increase predation by granivores. Functional trait‐dependent seed predation might act as an equalizing effect for species coexistence, that is, it levels the competition among plant species by allowing seeds with lower germination rates to grow in the absence of the more competitive seeds (Larios et al., 2017; Stump & Chesson, 2017). Our results suggest that this coexistence mechanism is likely at play because the most removed seeds had functional traits advantageous for germination but were also more attractive for rodent consumption. The created dissimilarity in diversity patterns can also act as a stabilizing mechanism of species coexistence: with different abundances of resource species left in different landscapes, there is an increase in intra‐specific competition rather than inter‐specific, as same species have to compete for the same niche. Experiments using DivGUD can provide more insights into these coexistence mechanisms, while also informing on nonconsumptive cascading effects of perceived predation risk in foragers. This measurement can also be used to understand possible bottom‐up feedbacks, as the resource species biodiversity left behind by foragers can eventually shape the growth and diversity of the vegetation (Riginos & Grace, 2008).

Forager species richness did not vary with vegetation height; therefore, our results are not simply driven by variation in the forager community composition. Furthermore, despite conducting the experiment across 3 years, we did not detect much evidence for annual variation. Only the number of visits at patches differed between years, which was likely due to differences in sampled vegetation heights (in 2017 we could not sample patches higher than 70 cm). This indicates that neither environmental factors nor population density variation among years affected our results.

But—in addition to vegetation height—the number of forager species present had an effect on GUD and DivGUD (both $\bar{\alpha }$ and γ). This pattern was contrary to our expectations (prediction iv), as all rodent species were expected to react in a similar manner in safer landscapes (i.e., feed on the same resources), or that inter‐species interactions would exclude less competitive species from feeding, and thus not have an additional effect on the resources left. It is possible that some species have a greater effect on GUD/DivGUD, as species might have different foraging strategies (Thompson, 1982), activity patterns (Kołakowski et al., 2018), or learning behavior (Haupt et al., 2010). In our data, the minimum recorded activity of rodents occurred mostly in medium and high vegetation height categories (Figure 5, Figure S2), likely due to microhabitat heterogeneity. The yellow‐necked mouse (*A. flavicollis*) was most frequently recorded in the foraging patches, especially at medium vegetation height, an expected result given dominant habitat presence and behavior in relation to other species (Grüm & Bujalska, 2000; Hille & Mortelliti, 2011).

The unpredictability of foragers is common in experiments done in the wild since there is variation in the diversity of species, their respective abundances, potential among‐individual variation in states (e.g., starved individuals), age or experience (Bedoya‐Perez et al., 2013). Despite our artificial setting of equally profitable patches, these factors may have created some variation in the final giving‐up densities and biodiversity of resources. However, our results consistently indicated the importance of perceived predation risk, since we found similar effects of vegetation height on resource diversity (DivGUD). Future experiments should take into account the variation in the forager's species community, and how each species contributes to changes in DivGUD across landscapes of fear, further linking behavioral ecology with community ecology across trophic levels.

## 5 CONCLUSIONS

Foraging under perceived risk has cascading effects on resource species diversity at local and regional spatial scales, which can be measured via diversity at giving‐up density (DivGUD; Eccard et al., 2022). Thus, nonconsumptive predation effects can promote resource species coexistence in the landscape of fear of a forager with just the perceived predation risk shaping the forager‐resources interactions. Combining several food resource species of different functional traits into experimental assemblages provided a first glimpse into how perceived risk during foraging might modify coexistence mechanisms at the local and regional spatial scale. We hope that this experimental approach can pave the way to further studies on possible bottom‐up effects, such as the growth of plant species caused by differential feeding and scatter‐hoarding behavior of rodents. The changes in biodiversity occurring with the variation of fear become important when dealing with anthropogenic impacts or species reintroductions, which can further cascade through trophic networks or generate bottom‐up effects into other trophic levels.

## CONFLICT OF INTEREST

The authors report no conflict of interest.

## AUTHOR CONTRIBUTIONS

**Clara Mendes Ferreira:** Conceptualization (equal); data curation (equal); formal analysis (equal); methodology (equal); supervision (equal); validation (equal); writing – original draft (lead); writing – review and editing (equal). **Melanie Dammhahn:** Conceptualization (equal); methodology (equal); supervision (supporting); validation (supporting); writing – original draft (equal); writing – review and editing (equal). **Jana A. Eccard:** Conceptualization (equal); data curation (equal); formal analysis (equal); methodology (equal); supervision (lead); validation (equal); writing – original draft (equal); writing – review and editing (equal).

## Supporting information


Figures S1‐S2

## Data Availability

The data that support the findings of this study are openly available in OSF at https://doi.org/10.17605/OSF.IO/BKN3T.
