# Supplementary material for: Forager‐mediated cascading effects on food resource species diversity
Source: Ecol Evol. 2022 Nov 18;12(11):e9523. doi: 10.1002/ece3.9523 (PMC9674471; doi:10.1002/ece3.9523)
Supplement: Supplementary file 1 — Figures S1‐S2 [file ECE3-12-e9523-s001.docx]

**Forager-mediated cascading effects on food resource species diversity**

Authors: Clara Mendes Ferreira^1^, Melanie Dammhahn^2^, Jana Eccard^1^

1 - Animal Ecology, Institute for Biochemistry and Biology, University of Potsdam, Potsdam, Germany

2 - Behavioural Biology, Institute for Neurobiology and Behavioural Biology, University of Münster, Germany


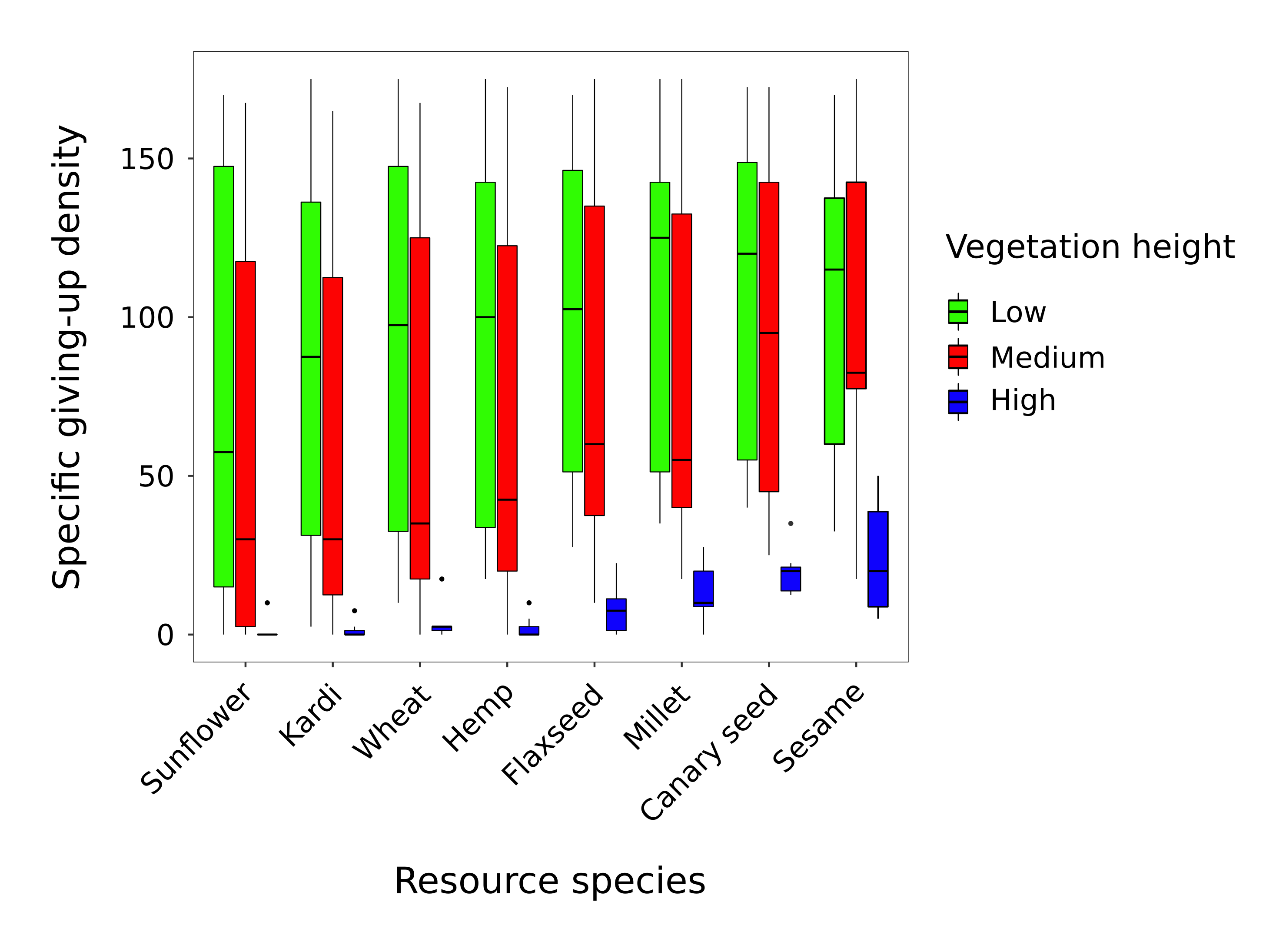


**Supplemental figure 1** – Total quantity of each resource species left by foragers, at all foraging landscapes, in three vegetation height categories: low: < 15 cm (green); medium: ≥15 and ≤ 52 cm (red); high: > 52 cm (blue). Seeds species are sorted by size.


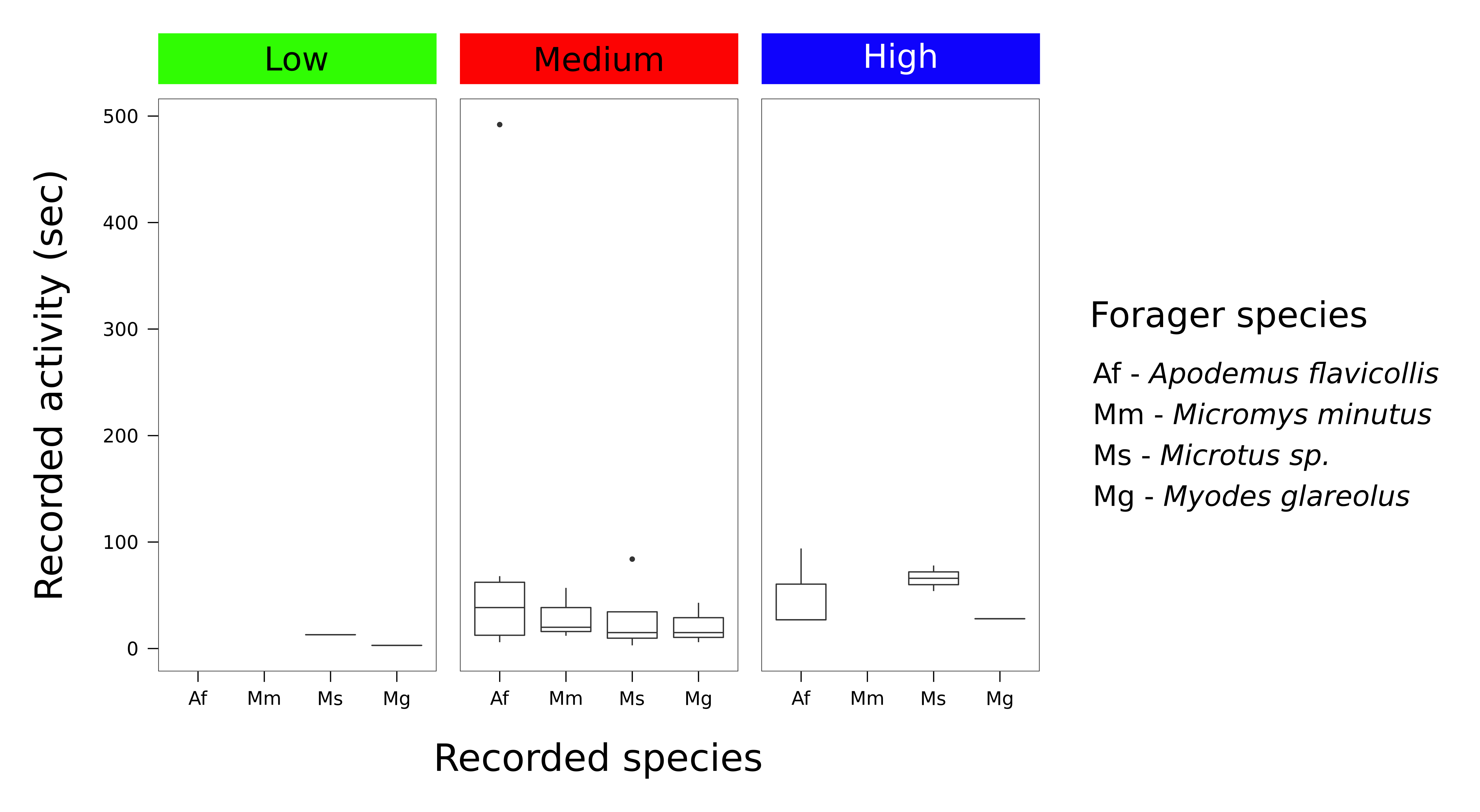


**Supplemental figure 2** – Minimum activity recorded by the camera traps for different forager species in three vegetation height categories: low: < 15 cm (green); medium: ≥15 and ≤ 52 cm (red); high: > 52 cm (blue). Activity was measured through photos taken by the camera traps, each photo consisted of one second of activity. Minimum recorded activity was summed for each landscape and species.
